# Does Sound Influence Perceived Duration of Visual Motion?

**DOI:** 10.3389/fpsyg.2021.751248

**Published:** 2021-12-02

**Authors:** Alessandro Carlini, Emmanuel Bigand

**Affiliations:** Laboratory for Research on Learning and Development, CNRS UMR 5022, University of Burgundy, Dijon, France

**Keywords:** motion, sound, pitch modulation, multimodal perception, internal models, time perception

## Abstract

Multimodal perception is a key factor in obtaining a rich and meaningful representation of the world. However, how each stimulus combines to determine the overall percept remains a matter of research. The present work investigates the effect of sound on the bimodal perception of motion. A visual moving target was presented to the participants, associated with a concurrent sound, in a time reproduction task. Particular attention was paid to the structure of both the auditory and the visual stimuli. Four different laws of motion were tested for the visual motion, one of which is biological. Nine different sound profiles were tested, from an easier constant sound to more variable and complex pitch profiles, always presented synchronously with motion. Participants’ responses show that constant sounds produce the worst duration estimation performance, even worse than the silent condition; more complex sounds, instead, guarantee significantly better performance. The structure of the visual stimulus and that of the auditory stimulus appear to condition the performance independently. Biological motion provides the best performance, while the motion featured by a constant-velocity profile provides the worst performance. Results clearly show that a concurrent sound influences the unified perception of motion; the type and magnitude of the bias depends on the structure of the sound stimulus. Contrary to expectations, the best performance is not generated by the simplest stimuli, but rather by more complex stimuli that are richer in information.

## Introduction

Multi-model perception is a crucial part of everyday life. This is notably the case when we look at an object producing sound and moving in the environment, like a car during a car race, for instance, where its sound is likely to contribute to the tracking of the visual target. In the same way, it seems easier to estimate how long was the solo of, let’s say, a saxophone player if we were both listening to the sounds and watching his/her movements. Both cases are an example of multi-modal perception, where visual and acoustic information are simultaneous. Indeed, the majority of our day-to-day perceptual experience is multi-modal. Each channel transmits information that contributes to – and influences – unified perception. However, to date, how the pieces of information combine remains a matter of debate.

In this paper, we focus on whether the perception of a visual event – a visual motion – may be influenced by the presence of concurrent auditory information. More specifically, we investigate the bimodal perception of the duration of a motion, paying particular attention to the structure of both the auditory and the visual stimuli. We defined different profiles for the stimuli of both modalities, from easier to more complex – and we tested different combinations of profiles, congruent and incongruent. In all proposed tests, the two stimuli (auditory and visual) were synchronous and provided the same information of duration; thus, perceptual differences found in participants’ responses are discussed considering both the type of information and the structure that characterize each stimulus.

### The Power of the Multi-Modal Perception

In the last 30 years, a large body of research has shown a variety of advantages offered by the combination of different senses, such as touch and vision (Heller, 1982; Lederman et al., 1986), touch, vision and olfaction (Dinh et al., 1999) or taste and vision (Bult et al., 2007). Interestingly, these results show that the perception in multi-modal conditions provides not only a larger amount of information but also significant improvements in the accomplishment of different tasks (Hommel and Zmigrod, 2013, for a review). For instance, Bahrick et al. (2004) reported that selective attention to relevant elements is facilitated by the overlapping of visual and auditory perception, and it is attenuated when the pertinent information is unimodal. In a spatial-localization task using auditory and visual stimuli, Teder-Sälejärvi et al. (2005) confirmed that responses to bimodal stimuli were faster and more accurate than unimodal stimuli (Giard and Peronnet, 1999). Frassinetti et al. (2002) showed a dynamic collaborative relationship between auditory and visual systems, in which the presence of an auditory stimulus enhances the efficiency of the visual system in a detection task.

Previous research has investigated simple capabilities such as the localization of stimuli, almost exclusively in stationary paradigms. Conversely, the present work focuses on the auditory-visual perception of a moving target. Sound and movement are dynamic stimuli, evolving through time. We investigate the possibility that the two dynamics may interfere, notably for the evaluation of duration. More specifically, we investigate if the features of each modality influences – and can alter – the unified perception of the motion duration.

### The Visual Perception of (Silent) Motion

The stimulus structure tunes the corresponding perception. Previous studies by Piaget and Werner (1958), and Runeson (1974) provided evidence that the seen-velocity of a moving object was not perceived consistently with its physical-velocity. According to Piaget, the perceived velocities at the beginning and the end of the visible trajectory are systematically overestimated. Similarly, Runeson reported that a constant velocity stimulus was erroneously perceived as slowing down during its trajectory. Interestingly, Runeson also demonstrated that it is possible to tune the perceived velocity of a stimulus by manipulating the structure of its velocity profile. Actis-Grosso and Stucchi (2003) conducted six spatial-localization experiments and presented a detailed analysis of the starting and ending point misperception. Consistently with the previous literature, their experiments revealed the role of the velocity profile in modulating the misperception of the motion itself.

Within the wide panorama of different types of motion, *biological motion* represents a special condition. Its kinematic profile particularly supports our capabilities to identify the agents, to identify the nature of movements, and to predict the movement evolution (Johansson, 1973; Hiris, 2007). Concerning the *temporal aspects* of visual motion perception, Brown (1995) used stationary and non-stationary targets to investigate the effect of velocity on the duration perception. Compared to the stationary condition, he found that the perceived duration of a moving stimulus is systematically overestimated and that this effect is stronger as the motion is faster. Previous research using the same family of velocity profiles that are used in the present work, has demonstrated that biological kinematics allows better performance for our perceptual system. When a motion follows the biological law of motion (LoM), we are able to better predict future motion, and better reconstruct a hidden past trajectory (Pozzo et al., 2006; Carlini et al., 2012). Moreover, Carlini and French (2014) tested different velocity profiles in a duration estimation task; their results revealed that, in performing the required task, motion hand-tracking is more effective than visual-tracking alone, but only if the target moves according to a biological LoM.

Some research has focused on the perception of motion duration, including biological motion, using different types of stimuli. Kaneko and Murakami adopted vertical Gabor patches as stationary and moving stimuli, and confirmed the dilation of perceived motion duration compared to the static condition. Their results also confirmed that higher velocities induce a higher magnitude effect, with stationary motion also (Kaneko and Murakami, 2009). Matthews presented rotating or translating shapes, in three conditions: constant speed, accelerating motion, and decelerating motion. Interestingly, he found that constant speed stimuli have the longest perceived duration (Matthews, 2011). Wang and Jiang investigated the perception of duration in the specific case of biological motion, using static and dynamic representations of a point-light walker. Their results showed that biological motion is perceived to be significantly longer than the corresponding static image. Moreover, authors showed that biological motion is perceived as significantly longer than non-biological conditions, regardless of whether the viewer recognized the biological nature of the stimulus (Wang and Jiang, 2012).

We are now interested in exploring what results can be achieved by coupling an acoustic stimulus to this type of kinematics.

### When a Sound Is Present

It is a common experience that the presence of music influences our perception of time. Generally speaking, compared to the silent condition, the presence of music shortens the perceived duration of a time interval (Hul et al., 1997; North et al., 1999; Gueguen and Jacob, 2002). However, the effect of the presence of music or sounds could be varied and complex. For instance, the duration of a melody is judged shorter than a non-melodic stimulus (Bueno and Ramos, 2007; Droit-Volet et al., 2010). The familiarity with the piece of music also contributes to shortening the perceived duration (Yalch and Spangenberg, 2000; Bailey and Areni, 2006). Concerning, more specifically, the presence of sound in multimodal perception, results from a large body of research have found auditory dominance over other modalities, both in time judgments (Repp and Penel, 2002; Burr et al., 2009; Grondin and McAuley, 2009; Grahn et al., 2011) and in spatial localization (Pick et al., 1969; Bertelson and Radeau, 1981; Radeau and Bertelson, 1987). Visual-auditory perception is probably the most studied multimodal condition (Leone and McCourt, 2015; Odegaard et al., 2015; Chen et al., 2016).

Perhaps one of the best known and most analyzed studies is the work of Colavita, which led to the identification of the Colavita Effect, a known condition of visual dominance (Colavita, 1974; Spence et al., 2012). In his experiment, the author presented to participants either an auditory (tone) or visual (static point light) stimulus, under mono- and bi-modal conditions, and found that in most bimodal trials participants perceived only the visual stimulus, neglecting the auditory stimulus. An analogous set of stimuli is often used to study the Ventriloquism Effect (Hartcher-O’Brien and Alais, 2011; Vidal, 2017), the induced visual motion (Soto-Faraco et al., 2002), and the induced auditory motion (Mateeff et al., 1985).

Research on induced motion obtained through some specific Ventriloquism Effect conditions provide us with interesting information. Freeman and Driver proposed an interesting paradigm, based on two bars that alternate between opposite hemispheres, generating the sensation of induced motion. Adding an acoustic stimulus, and leaving the visual stimulation unchanged, they showed that the variation of the timing of the acoustic stimuli strongly influenced the direction of the obtained induced motion (Freeman and Driver, 2008). Kafaligonul and Stoner further developed Freeman and Driver’s paradigm, and showed that the misalignment of acoustic stimuli, relative to a pair of visual stimuli, can affect not only the direction (Experiment 1), but also the velocity (Experiment 2) of induced motion (Kafaligonul and Stoner, 2010). More recently, Ogulmus et al. (2018) have added further evidence showing that the timing of the auditory stimulus can modulate the perception of the velocity of induced motion. They presented to participants a pair of auditory stimuli that are slightly temporally misaligned with respect to the visual flashes. When the auditory stimuli are shifted within the temporal interval separating the visual stimuli, the apparent motion was perceived to move faster than the condition in which the auditory stimuli are outside the same interval (Ogulmus et al., 2018).

Specifically investigating the real motion condition, Prime and Harris used a moving dot, produced by a laser pointer on a screen, and a pure tone diffused by a pair of audio speakers placed behind the screen, to investigate motion prediction capability (Prime and Harris, 2010). Patrick and Anderson explored the influence of auditory rhythm on visual motion on prediction, using a disk moving on a screen and a variable tonal structure (Patrick et al., 2021). Brooks et al. (2007) investigated the effect of auditory motion on visual perception of biological motion, using a set of moving point reproducing the biological pointlight walkers of Johansson (1973). In other research work regarding the audio-visual perception of motion, Alais and Burr investigated human threshold to perceive audio-visual motion, using moving stimuli in both visual (a set of moving dots on a screen) and auditory (modulating the sound between the left and right channels of audio speakers) modes (Alais and Burr, 2004). The team of Meyer and Wuerger investigated bimodal perception using a set of flashing LEDs and loudspeakers, equispaced in the azimuthal plane, and allowing motion to be reproduced to the left or right in the participant’s frontal space (Meyer et al., 2005; Wuerger et al., 2010).

In bimodal audio-visual condition, the behavioral and neurophysiological studies show activation of both corresponding brain cortices; more interestingly, they also reveal the existence and the activation of shared structures responsible for the multisensory processing (Keetels and Vroomen, 2007; Roach et al., 2011; Stevenson et al., 2012; Thelen et al., 2016).

### The Present Research

The perception of time is not isomorphic to physical time and can be distorted by several factors (Allan, 1979; Hancock and Weaver, 2005; Droit-Volet and Meck, 2007). Research has demonstrated that perception is not just physical data encoded by senses: the way and the context in which each stimulus comes, its temporal features, its structure, and the presence of other stimuli tune the resulting unified perception.

In everyday experience, moving stimuli are usually associated with other information, notably sounds. Sound and motion are dynamic structures, both having strong implications in the life of a human being. They provide information about the presence of other living beings, or about changes in the surrounding environment. In short: motion and sound are strongly -and very often causally- related. The sensitivity to biological motion, and the dominance of auditory perception, are strong evidence of their ancestral implication on survival. Analyzing these elements more closely, some questions naturally arise. Could a sound modify the perception of an observed motion? Is there a relationship between the structure of each percept and the duration estimation of the whole event? To our knowledge, no previous studies have addressed these points.

Here we propose an original paradigm trying to respond to these questions. A visual target moving on a screen and a concurrent sound were presented to participants, in a duration reproduction task. The two percepts that compose the stimulus had the same duration, and were therefore consistent with respect to the temporal estimation task demanded; however, each percept (motion and sound) had a different structure. Nine different sound conditions and four different kinematics were tested, distributed in the three different experiments. We adopted a paradigm based on modulated sounds as auditory stimulus - instead of music or impulsive sounds, as previous works did. The nine sound conditions have been designed to constitute an increasing scale in complexity, from the simplest constant sounds, up to the more complex profile of the bell-shaped sound (see section “Materials and Methods” paragraph for more details).

Generally speaking, we expect that the bimodal perception would improve the duration estimation accuracy, compared to the silent-motion condition (Heller, 1982; Bahrick et al., 2004; Teder-Sälejärvi et al., 2005; Hommel and Zmigrod, 2013). We also expect that the stimuli featured by a biological profile would permit a more accurate time estimation, according to the previous literature (Pozzo et al., 2006; Carlini and French, 2014). A principle of economy leads us to expect that a simpler stimulus may be easier to process, and thus provide better accuracy in duration perception.

Moreover, the most recent findings on multisensory integration lead us to expect that a correlated stimulus pair should result in a more accurate perception, and consequently a more accurate assessment of duration (Spence, 2011; Parise et al., 2012; Hommel and Zmigrod, 2013; Parise and Ernst, 2016). Findings of older research, instead, such as those of Piaget and Runeson previously presented, remind us that a single percept can induce a misperception and a dysregulation of the overall unified perception (Piaget and Werner, 1958; Runeson, 1974; Actis-Grosso and Stucchi, 2003). The aim of the present work is to contribute to the comprehension of these mechanisms of perceptual integration.

## Experiment 1: Does Adding a Simple Sound Improve Motion Perception?

In Experiment 1, we assessed whether participants are better able to estimate the duration of a moving visual stimulus when the motion is combined with a constant sound. In short, this first experiment, compares the “silent” mono-modal to two bimodal perception conditions. Participants were asked to evaluate the duration of a moving visual target, both in silent condition and in sound conditions. In sound conditions, the target motion was associated with the sounding of a stable-sine wave, either at 440 Hz or at 880 Hz. To verify the possible interaction between the structures of stimuli, four different kinematics were tested and imposed on the target motion: one “biological,” and three non-biological. In agreement with the results of previous works (Pozzo et al., 2006; Carlini and French, 2014), we expect that biological kinematics would ensure more accurate responses, compared to the non-biological motion conditions.

### Participants

Twenty students and employees of the University of Burgundy took part in the experiment (10m, 10f, age: *M* = 23.15, *SD* = 5.58). All reported having normal or corrected-to-normal visual acuity in addition to normal binaural hearing. All participants were naive with regard to the purpose of the study. The protocol was in agreement with the Helsinki Declaration (1964 and subsequent revisions), the CNRS’s guidelines (French National Center for Scientific Research), and the French Psychology Society Code of Conduct. The protocol was non-invasive and posed no risks for participants. French legislation and the directives of the University of Burgundy do not require approval by an ethics review board for this type of research. A qualified person supervised the research. Written informed consent was obtained from each participant in the study.

### Apparatus and Stimuli

All conditions were composed of a visual moving target and an auditory sound (except for the “silent” condition). The visual target consisted of a light-gray disk (luminance ∼70 cd/m^2^), vertically moving on a black background (approximately luminance ∼2.0 cd/m2); the auditory stimulus consisted of a constant frequency sound (about 50 dB), timely coupled with the visual stimulus. In the silent condition, only the visual stimulus was presented. Participants were asked to reproduce the perceived duration of the motion, at each presentation. All stimuli were created and displayed using Matlab (The MathWorks, Inc.) and Psychophysics Toolbox for Matlab (Brainard, 1997; Pelli, 1997). Visual stimuli were displayed on a Dell 17-inch color LCD monitor (1280 × 1024, 60 Hz refresh rate). The auditory stimuli were generated by a Sound Blaster Audigy 2 ZS sound card (Creative Technology, Ltd), and a Z623 speaker system (Logitech Ltd); speakers were positioned behind the monitor, to obtain the perception that sound originated from the moving target (Perceptual fusion effect: Howard and Templeton, 1966; Bertelson and Radeau, 1981; Morein-Zamir et al., 2003). Participants sat at a comfortable viewing distance from the screen (about 60 cm). A push-button was situated in front of the screen at a comfortable distance (about 30 cm from the participant) to allow participants to give the response. The experiment was performed in a dimly lighted and soundproof room.

The visual stimulus (a light-gray disk, fifteen pixels in diameter, about 0.38° in participant’s visual angle) moved vertically within a presentation window of 1000 × 740 pixels centered on the screen. Each trial was randomly displayed in a different position within the presentation window.

**FIGURE 1:**
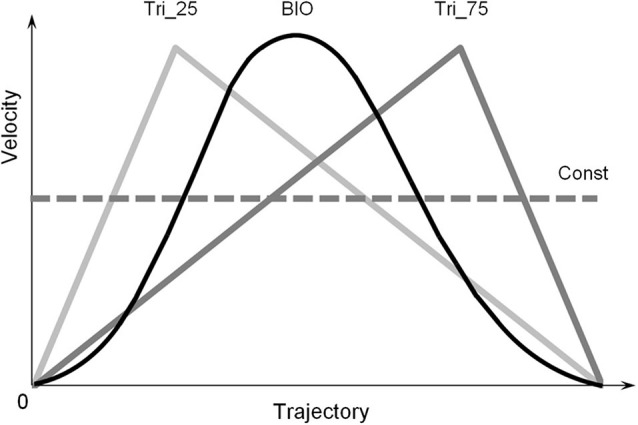
Law of Motion profiles. The four velocity-profiles adopted in the three experiments: one “Biological” velocity profile (“BIO,” black line), two “Triangular” profiles (“Tri_25” and “Tri_75,” gray lines) and one Constant profile (“Const,” dotted line). The biological velocity profile was obtained as the average of several arm-pointing movements, upward oriented and performed with a straight arm, previously recorded in frontal view. The two triangular velocity profiles are characterized by linear acceleration and deceleration; they differ in the position of the peak of velocity: at 25% of the trajectory for the Tri_25, and 75% of the trajectory for the Tri_75. The Const velocity profile consisted of a constant-velocity movement over the whole trajectory. The four profiles are characterized by the same average velocity. The four different durations of the motion (0.5, 1.0, 1.5, or 3.0 s) correspond to the four average velocities: 30, 15, 10, or 5 [degrees/second] for participants.

For all trials, the length of the trajectory was 600 pixels (about 15 degrees in the participant’s visual field). The visual stimulus always moved upward in a straight vertical line; it moved accordingly to one of the four different Laws of Motion (LoM), one “biological” and three non-biological (Figure 1). The biological velocity profile (“BIO”) corresponds to a generalized hand pointing movement, with a straight arm and finger. It was obtained as an average velocity profile of several arm-pointing movements, upward oriented, previously recorded in frontal view (Papaxanthis et al., 1998). A peculiar characteristic of the upward BIO velocity profile is the peak in velocity at 45% of the trajectory. The three non-biological laws of motion consisted of one constant velocity profile (“Const”) and two triangular velocity profiles, both characterized by linear acceleration and deceleration and a peak of velocity at 25% of the trajectory (“Tri_25”), or 75% of the trajectory (“Tri_75”), respectively. These adopted laws of motion were chosen from those already used in previous research (Papaxanthis et al., 1998; Pozzo et al., 2006; Carlini et al., 2012; Carlini and French, 2014), and selected to compare the effects of fundamental motion profiles such as constant motion, uniformly accelerated/decelerated, and biological motion.

In each trial, the onset and offset of the auditory stimulus were synchronized to the onset and offset of the motion display. Therefore, the two stimuli were always concurrent and featured by the same duration. In this first experiment, the auditory stimuli consisted of two constant frequency sounds, an “A4” and an “A5” note (440 and 880 Hz tone, respectively), plus the silent condition.

**FIGURE 2:**
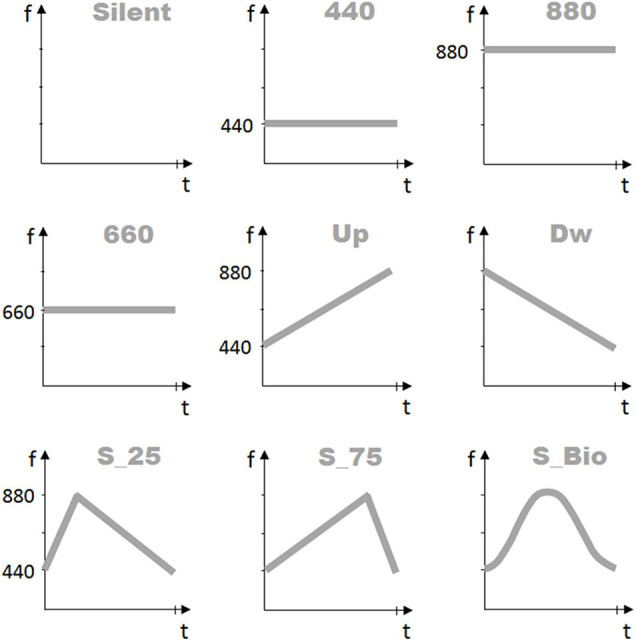
Sound frequency profiles. The nine graphs represent the frequency profiles of the auditory stimuli proposed to the participants in the three Experiments. The first line presents the three auditory conditions proposed in Experiment 1: “Silent” (no-sound), “440” (constant sound frequency, at 440 Hz), and “880” (constant sound frequency, at 880 Hz). The second line presents the three auditory conditions proposed in Experiment 2: “660” (constant sound frequency, at 660 Hz), “UP” (linearly increasing frequency between 440 Hz and 880 Hz), and “DW” (linearly decreasing frequency between 880 and 440 Hz). Finally, the third line presents the three auditory conditions proposed in Experiment 3: “S_Tri_25” (triangular profile, between 440 and 880 Hz, peak at 25% of the total duration), “S_Tri_75” (triangular profile, between 440 Hz and 880 Hz, peak at 75% of the total duration), and “S_BIO” (reproducing the bell shape of the “BIOlogical” velocity profile). *X*-axis represents the time duration of the stimulus, in seconds; *Y*-axis presents the sound frequency values in Hz.

Figure 2 presents the auditory features of each condition. Each motion condition, and concurrent sound, were presented in one of four possible durations (“Time” duration factor): 0.5, 1.0, 1.5, or 3.0 s. Each possible combination of the forty-eight conditions (4 LoM × 3 Sound × 4 Time durations) was presented four times. In total, 192 trials were presented in random order. The whole procedure took about 25 min.

### Procedure

Participants were informed about the nature of the test and that it was composed of two phases; oral and written information was provided before each phase. Participants performed the test always in the same order: (1) the pre-experiment phases and (2) the experiment phase.

In both phases, participants were asked to give the response, in each trial, by pushing and holding the button with his dominant hand, for a duration equal to the perceived displayed motion. They were expressly instructed to respond as accurately as possible. No time-constraints were given concerning the response. Each participant was clearly informed that the reproduction task was based on visual motion, and the pre-test allowed them to become familiar with this procedure. In the debriefing performed after each test, all participants confirmed that they interpreted the task correctly.

#### Pre-experiment

The pre-experiment aimed to familiarize participants with button-pressing to reproduce the previously perceived time interval. The results of this phase also constituted the base-line for the responses of the following experimental phase. Each participant started this first phase by pressing the push-button. A static visual stimulus (a light-green disk, 30 mm in diameter) was presented at the center of the screen, for a pre-determined duration. Sixteen different durations were possible, randomly selected between 0.4 and 3.4 s, interspaced by 0.2 s. The participant was asked to reproduce, after each disk disappearance, the perceived display duration by pushing and holding down the push-button for an equivalent time interval. The release of the push-button started the next trial, spaced by a blank interval lasting 1.0 ∼ 2.2 s. The pre-experiment lasted approximately 3 min. After completion of this first phase, each participant was automatically introduced to the test phase.

#### Experiment

When ready, each participant started the experiment by pressing the push-button. Each trial began with an initial blank interval lasting 1.0 ∼ 2.2 s; then the target (the light-gray disk, 15 pixels diameter) became visible and moved at the same time, and disappeared at the end of its motion. Before the moving target’s appearance, a light-gray dot flashed once for 150 ms, at the point where the moving stimulus would appear and started moving. The sound stimulus was played synchronously with the moving target, from the beginning to the end of the motion. After the disk disappearance, each participant gave his or her responses by holding down the press-button, for the amount of time her/he judged equivalent to the perceived duration of the motion. The release of the push-button started the new trial. At the end of the experiment, a debriefing was provided to the participants.

### Results and Discussion

Participants’ responses were evaluated by Constant Error and Variable Error. Constant Error (CE) is the mean of the participant’s estimation errors in each condition, and corresponds to the inverse of the accuracy; Variable Error (VE) is the standard deviation of the participant’s estimation errors and corresponds to the inverse of precision. The standard deviation of CE expresses the inter-subject variability, where VE expresses the intra-subject variability. All the estimation errors were obtained by subtracting the actual stimulus duration from the participant’s estimations, thus positive values indicate over-estimations and negative values indicate under-estimations of the time. All time values are expressed in seconds.

We performed a multi-way ANOVA on CEs on the three within-subjects main factors: Sound (Silent, 440, 880), Law of Motion “LoM” (BIO, Tri_25, Tri_75, Const), and Time (T1 = 0.5, T2 = 1.0, T3 = 1.5, T4 = 3.0 s). The same three within factors were employed to perform the one-way ANOVAs on VEs. Statistical significance was fixed at *p* < 0.05.

The ANOVA analysis on CE showed the Sound factor as statistically significant [*F*(2,38) = 14.324, *p* < 0.0001, η^2^ = 0.43]. Participants were more accurate in the Silent condition, and a Tukey HSD *post hoc* confirmed the significance of the difference between Silent and 440 (*p* = 0.001), and between Silent and 880 (*p* = 0.005). The difference between 440 and 880 appeared not-significant (*p* = 0.17).

ANOVA showed the LoM factor also as statistically significant [*F*(3,57) = 23.430, *p* < 0.00001, η^2^ = 0.55]. As expected, the BIO kinematics appeared the most effective condition, and the Tukey HSD *post hoc* analysis confirmed the difference between BIO and Tri_75 (*p* = 0.0047), and between BIO and Const (*p* = 0.0001). Instead, the constant motion (“Const” condition) appeared the worst condition, and the Tukey HSD *post hoc* analysis confirmed the significance of the difference between Const and all the other conditions (*p* < 0.003).

**FIGURE 3:**
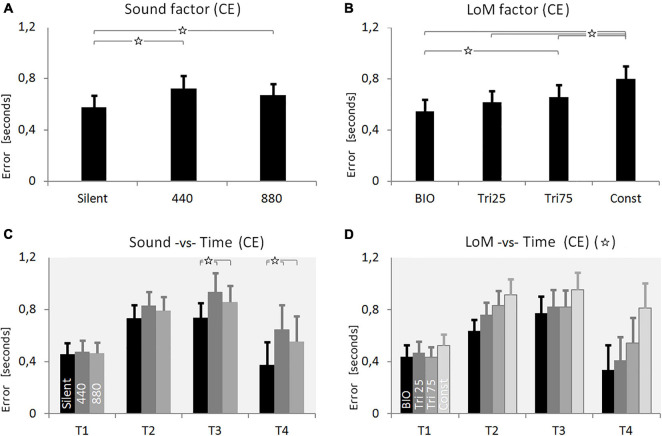
Results of Experiment 1. **(A)** Constant Error (CE) for the three Sound conditions: Silent, constant frequency at 440 Hz, constant frequency at 880 Hz (pitch envelopes are presented in Figure 2 and described in the text). **(B)** CE for the four Laws of Motion (LoM): Biological velocity profile (BIO), Triangular velocity profile with a peak of velocity at 25% of the trajectory (Tri25), Triangular profile with a peak at 75% (Tri75), Constant velocity profile (Const). **(C)** Interaction between Sound and Time main factors. Each group of bars refers to a different Time condition (T1 = 0.5 s, T2 = 1.0 s, T3 = 1.5 s, T4 = 3.0 s); in each group, bars respectively presents the CE value of the three Sound conditions: Silent, 440, 880 (respectively in black, dark-gray, and light-gray color). Sound characteristics and statistical significances are described in the text. **(D)** Interaction between LoM and Time main factors. For each Time conditions (T1 = 0.5 s, T2 = 1.0, T3 = 1.5, T4 = 3.0) bars represent the CE of the LoM conditions (from the black to the light-gray bar, respectively: BIO, Tri25, Tri75, Const). Statistical significances are described in the text. For all charts: *Y*-values are in seconds, error bars represent standard errors of the mean, and stars indicate significant statistical differences (when the star is in the headline, please refer to the text for details regarding the statistical differences between conditions).

The Time factor also appeared statistically significant [*F*(3,57) = 3.0796, *p* = 0.0345, η^2^ = 0.14]. As reported in Table 1 and graphically presented in Figure 3, the CE values increased from 0.47 s in T1 up to 0.84 s in T3, and then decreased again to 0.5 s in the T4 condition. The Tukey HSD *post hoc* failed to find the significant factors, but the LSD *post hoc* showed a significant difference between T1 and T2 (*p* = 0.038), T1 and T3 (*p* = 0.015), and T3 and T4 (*p* = 0.039).

**TABLE 1:** Results of Experiment 1 – Constant Error (CE) and Variable Error (VE) for the three main factors: Sound, Law of Motion (LoM), and Time.

Sound:	Silent	440	880
	CE = 0.58 ± 0.41	CE = 0.72 ± 0.44	CE = 0.69 ± 0.39
	VE = 0.63 ± 0.21	VE = 0.60 ± 0.27	VE = 0.61 ± 0.25

LoM:	BIO	Tri_25	Tri_75	Const
	CE = 0.55 ± 0.40	CE = 0.62 ± 0.40	CE = 0.66 ± 0.42	CE = 0.80 ± 0.44
	VE = 0.61 ± 0.23	VE = 0.59 ± 0.24	VE = 0.61 ± 0.25	VE = 0.61 ± 0.27

Time:	T1	T2	T3	T4
	CE = 0.47 ± 0.37	CE = 0.79 ± 0.44	CE = 0.84 ± 0.57	CE = 0.52 ± 0.83
	VE = 0.29 ± 0.14	VE = 0.44 ± 0.19	VE = 0.46 ± 0.19	VE = 0.62 ± 0.22

*All values are given in seconds (mean value ± standard deviation).*

The ANOVA on CE revealed as significant all the interactions between the three main factors. The Tukey *post hoc* analysis on the interaction between Sound and LoM [*F*(6,114) = 7.4631, *p* < 0.00001, η^2^ = 0.28] revealed that the difference between Silent and the two constant sound conditions was significant for all the Laws of Motion except for the Const condition. Concerning the interaction between Sound and Time [*F*(3, 57) = 23.430, *p* = 0.0046, η^2^ = 0.17] the *post hoc* revealed the difference between Silent and constant sounds as not-significant in T1 and T2; instead, the difference became significant in T3 and T4 (*p* < 0.002). Finally, concerning the interaction between LoM and Time [*F*(9,171) = 6.2678, *p* < 0.00001, η^2^ = 0.25] the Tukey *post hoc* confirmed, for both the BIO and the Const LoM, the statistical difference from all other laws of motion in T2, T3, and T4.

The ANOVA on VE showed only the Time factor as significant [*F*(3,57) = 16.7407, *p* < 0.00001, η^2^ = 0.55]. The mean values of VE increased monotonically from T1 to T4; a Tukey HSD *post hoc* showed as significant the difference between T1 and all the other conditions, and between T4 and all the other conditions.

The numerical values resulting from the first experiment, for each main factor and condition, are summarized in Table 1 (mean values and standard deviations) both for CE and VE. As a first result, it is possible to notice a systematic overestimation of the motion duration. The comparison between auditory conditions shows that the presence of a constant sound induces a larger error in the participants’ responses, compared to the silent condition (Figure 3A). The silent condition guarantees an estimation error 18% smaller than the constant sounds; the *post hoc* test shows the difference between silent and constant sound conditions is statistically significant. Contrarily to the initial expectations, this result suggests that the presence of a constant sound does not improve our performance in estimating the motion duration. The statistical differences between the two constant sound conditions appear non-significant, suggesting that – in the tested conditions – differences in pitch do not induce differences in perception of duration.

Concerning the main factor LoM, as expected, more accurate estimations were possible when the target moved accordingly to the biological kinematics. Moreover, it appears interesting that participants committed the largest errors with the constant-velocity motion, which appears to be the motion with the simplest kinematics. The benefit of the BIO kinematics and the low accuracy originated by the Constant motion became more evident when we consider the interaction between LoM and Time factors (Figure 3D). The low accuracy shown by participants with the constant velocity motion leads to speculation as to whether this result was due to the simple nature of the stimulus itself. The main feature of the constant motion is the absence of variations for the entire motion. Consequently, it appears possible that the absence of variation in the stimulus deprives the percept of references useful for perceptual subdivision and time estimation.

In this first Experiment, the relation between LoM and Sound appears to be statistically significant. The *post hoc* shows that only the Constant motion is statistically different from the other three kinematics, in all Sound conditions. Interestingly, in the Silent condition, the differences between all the Laws of Motion became significant; it is possible to suppose that the presence of sound (a constant sound, in this case) would blur the differences among kinematics.

Finally, in the conditions of longer duration (especially in T4), the differences appear even more pronounced, both between the different sound conditions (Figure 3C), and between the different laws of motion (Figure 3D).

## Experiment 2: Is the Performance Improved by Variable Sounds?

The first experiment showed that the presence of a constant sound increases the CE in estimating the motion duration, compared to the silent condition. This result appeared in contrast with previous findings, which generally report an enhancement in multi-modal conditions.

Two factors deserve attention since they might influence the participants’ responses: (1) the presence of variations within the stimuli, and (2) the similarity/dissimilarity between sound and motion profiles. As previously indicated, indeed, the coherence between visual and acoustic stimuli can improve perceptual performance, whereas the incoherence would create a “perceptual interference,” thus originating worse duration estimates.

The aim of the second experiment is to introduce a dynamics in the auditory stimulus, to test whether a changing tone, coupled with the visual motion, can modulate its perceived duration. To this purpose, we compared (i) a constant frequency sound to (ii) a linearly increasing frequency and (iii) a linearly decreasing frequency sounds. We chose a linear increasing and decreasing of the pitch, for this second experiment, because they represent the simplest continuous variation of a sound.

### Participants

Twenty-one new participants, students and employees of the University of Burgundy, took part in the experiment (5m, 16f, age: *M* = 22.19, *SD* = 5.03). All reported having normal or corrected-to-normal visual acuity in addition to normal binaural hearing. All participants were naive with regard to the purpose of the study. The protocol presented to participants is the same as described and used in the first experiment. All participants in the study gave written informed consent.

### Apparatus, Stimuli, and Procedure

Laws of Motion and Time durations were the same as adopted in the first experiment. The three Sound conditions consisted of two variable sounds and a constant-frequency sound (Figure 2, second line). The two variable sounds consisted of a linearly increasing frequency sound (labeled as “UP”) and a linearly decreasing frequency sound (labeled as “DW”), both varying within the interval 440 Hz ÷ 880 Hz. We compared these two variable sounds to a 660 Hz constant frequency sound so that the three auditory stimuli present the same average frequency. The 192 trials (4 LoM × 3 Sound × 4 Time × 4 repetitions) were presented in random order; the whole procedure took about 25 min.

Apparatus and procedure were the same as described in Experiment 1.

### Results and Discussion

The analysis of the collected data was conducted accordingly to the same criteria already described in the first experiment. One participant gave responses more than twice the standard deviation from the mean of the group and his data were removed from the analyses.

**FIGURE 4:**
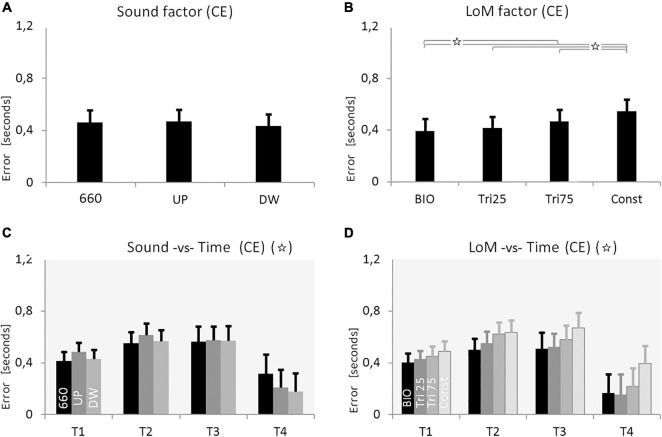
Results of Experiment 2. **(A)** Constant Error (CE) for the three Sound conditions: constant frequency at 660 Hz, increasing pitch sound (UP), decreasing pitch sound (DW) (pitch envelopes are presented in Figure 2 and described in the text). **(B)** CE for the four Laws of Motion (LoM): Biologic velocity profile (BIO), Triangular velocity profile with a peak of velocity at 25% of the trajectory (Tri25), Triangular profile with a peak at 75% (Tri75), Constant velocity profile (Const). **(C)** Interaction between Sound and Time main factors. Each group of bars refers to a different Time condition (T1 = 0.5 s, T2 = 1.0 s, T3 = 1.5 s, T4 = 3.0 s); in each group, bars respectively presents the CE value of the three Sound conditions: 660, UP, DW (respectively black, dark-gray, and light-gray color). Sound characteristics and statistical significances are described in the text. **(D)** Interaction between LoM and Time main actors. For each Time conditions (T1 = 0.5 s, T2 = 1.0, T3 = 1.5, T4 = 3.0) bars represent the CE of the LoM conditions (from the black to the light-gray bar, respectively: BIO, Tri25, Tri75, Const). Statistical significances are described in the text. For all charts: *Y*-values are in seconds, error bars represent standard errors of the mean, and stars indicate significant statistical differences (when the star is in the headline, please refer to the text for details regarding the statistical differences between conditions).

The ANOVA analysis was performed on CE and VE, involving the three within main factors: Sound (660, UP, DW), Law of Motion (BIO, Tri_25, Tri_75, Const), and Time (T1 = 0.5 s, T2 = 1.0, T3 = 1.5, T4 = 3.0). Statistical significance was set at *p* < 0.05. The Sound main factor alone appeared not significant; however, when we considered both Sound and Time factors, the interaction appears statistically significant [*F*(6,114) = 3.8604, *p* = 0.0015, η^2^ = 0.17]. As presented in Figure 4, the three Sound conditions appeared equivalent in the shortest time durations. Instead, in the longest Time condition (T4 = 3.0 s), the variable sounds permitted an estimation error significantly smaller than the constant sound (mean values ± SD, in seconds: CE_660___*T*__4_ = 0.31 ± 0.15; CE_*UP_T*__4_ = 0.21 ± 0.13; CE_*DW_T*__4_ = 0.18 ± 0.13). We performed a Tukey HSD *post hoc* analysis between each pair of conditions. Results show that the statistical difference between Constant and UP sounds only approaches the significance (*p* = 0.064), whereas the statistical difference between Constant and DW sounds is largely significant (*p* = 0.004). The same *post hoc* showed the results of the two variable sounds as statistically equivalent (*p* = 0.999).

Also the LoM main factor appeared statistically significant [*F*(3,57) = 12.683, *p* < 0.00001, η^2^ = 0.40]. As expected, BIO kinematics supported the best accuracy; again, the constant motion originated the worst performance. The Tukey HSD *post hoc* revealed a significant difference between the Constant motion and all the other conditions, and between BIO and both Tri_75 and Const condition. Finally, the multi-way ANOVA revealed as significant also the Time factor [*F*(3,57) = 5.7054, *p* = 0.0017, η^2^ = 0.23], and a Tukey HSD *post hoc* showed as significant the differences between T4 and the other three Time conditions.

In this second experiment, we also found a systematic overestimation of the duration in all conditions. A first examination of the mean CE values leads to suppose that the three Sound conditions were equal (Table 2). However, the analysis of the interaction between Sound and Time revealed a large difference between constant sound and variable sounds at the maximum duration condition (T4 = 3.0 s). More specifically, in T4 the variable sounds guaranteed a more accurate time estimation, whereas the constant sound produced a significantly larger error (the Constant sound condition originates a CE of 40% larger than the variable sound conditions; see Figure 4C).

**TABLE 2:** Results of Experiment 2 – Constant Error (CE) and Variable Error (VE) for the three main factors: Sound, Time, and Law of Motion (LoM).

Sound:	660	UP	DW
	CE = 0.46 ± 0.42	CE = 0.47 ± 0.40	CE = 0.44 ± 0.39
	VE = 0.49 ± 0.15	VE = 0.50 ± 0.13	VE = 0.51 ± 0.14

LoM:	BIO	Tri_25	Tri_75	Const
	CE = 0.39 ± 0.41	CE = 0.42 ± 0.39	CE = 0.47 ± 0.41	CE = 0.55 ± 0.41
	VE = 0.50 ± 0.15	VE = 0.50 ± 0.14	VE = 0.49 ± 0.14	VE = 0.48 ± 0.15

Time:	T1	T2	T3	T4
	CE = 0.44 ± 0.32	CE = 0.58 ± 0.39	CE = 0.57 ± 0.51	CE = 0.23 ± 0.63
	VE = 0.27 ± 0.12	VE = 0.36 ± 0.13	VE = 0.40 ± 0.15	VE = 0.56 ± 0.15

*All values are given in seconds (mean value ± standard deviation).*

Concerning the LoM main factor, the results were equivalent to the first experiment: the BIO kinematics guaranteed the best estimation, whereas the Const condition generated the largest error. For the Sound main factor, as well as for the LoM main factor, the differences among the four conditions become more evident when considering the longer durations of the stimulus (Figures 4B,D).

The two variable sound conditions UP and DW appeared statistically equivalent, suggesting that the relationship between the direction of the target motion and the direction of the sound variation does not play a relevant role in the present task. Finally, no significant interactions were found between Sound and Law of Motion; the auditory stimulation produce equal effects regardless of the kinematic type, and vice versa.

These results suggest that in each perceptual channel, the presence and the structure of a stimulus are able to define (and bias) the unified perception, but in an independent manner. For instance, the presence of a sound does not disrupt the helpful support provided by the biological kinematics.

## Experiment 3: The Bio Shaped Sound Profile

In the first experiment, we compared the silent condition with two constant sound conditions; in the second experiment, we compared a constant sound with two linearly variable sounds. In both experiments, we found that the sound influences the perception of time. Moreover, results show that: (i) compared to the silent condition, the presence of a constant sound leads to a decrease in performance for the perception of duration; and (ii) compared to a constant sound, the presence of an increasing or decreasing sound improves the perception of duration. To account for these results, we suggest that – not only the presence of a sound – but especially the structure of the auditory stimulus plays a relevant role in the definition of the unified perception, and the estimation of duration.

The structure of the auditory stimulus in previous experiments was extremely simple: the pitch profile was constant in the first experiment, and linearly increasing and decreasing in the second experiment. The purpose of this third experiment is to test whether a more complex melodic contour can originate an even stronger influence on the unified perception.

Based on the hypothesis that the variations in the stimulus structure may play a favorable role in perception, we expect that more structured sounds would better support timing, and consequently the estimation of motion duration would be more accurate (and possibly even more precise).

In this third experiment, we implemented three new sound stimuli, whose pitch profile has the same shape as the velocity profile of the three non-constant motions. Figure 2 presents the three sound profiles, in the third line. The first two profiles were triangular-shaped, composed of a linear increasing-decreasing sound frequency (labeled “S_Tri_25” and “S_Tri_75,” where the “S” specifies a Sound). The third sound profile adopted the same bell-shaped profile of the biological motion; this sound profile (labeled “S_BIO”) was featured by a non-linear pitch variation and represented the more complex auditory condition.

Since the biological profile has shown to provide particular support for visual perception, we expect that the S_BIO condition would produce the best performance in the duration estimation task.

### Participants

Twenty-one new participants, students and employees of the University of Burgundy, took part in the experiment (6m, 15f, age: *M* = 21.57, *SD* = 3.50). All reported having normal or corrected-to-normal visual acuity in addition to normal binaural hearing. All participants were naive with regard to the purpose of the study. The protocol presented to participants is the same as described and used in the first experiment. Written informed consent was obtained from each participant in the study.

### Apparatus, Stimuli, and Procedure

The three Laws of Motion and the four Time durations were the same as adopted in the previous experiments. The three Sound conditions were constituted by two triangular-shaped sounds and a bell-shaped sound, all starting and ending at the 440 Hz sound frequency (Figure 2). The two triangular-shaped sounds presented a peak-of-frequency at 880Hz, achieved at 25% of the stimulus length (S_Tri_25) or 75% of the stimulus length (S_Tri_75). The bell-shaped sound also presents a peak-of-frequency at 880Hz, positioned at 45% of the stimulus length. The 192 trials (4 LoM × 3 Sound × 4 Time × 4 repetitions) were presented in random order. The whole procedure took about 25 min.

The apparatus and procedure were the same as in the previous experiments.

### Results and Discussion

The analysis of the collected data was performed on the same criteria already described in the previous experiments. One participant gave responses more than twice the standard deviation from the mean of the group and his data were removed from the analyses.

The ANOVA analysis was performed of both CE and VE, based on the three within factors: Sound (S_Tri_25, S_Tri_75, S_BIO), Time (T1 = 0.5 s, T2 = 1.0, T3 = 1.5, T4 = 3.0), and Law of Motion (BIO, Tri_25, Tri_75, Const). This analysis of CE showed the Sound factor as statistically significant. The S_BIO condition appears supporting the best accuracy in the duration estimation, originating a CE 10% smaller than the average of the other conditions [*F*(2,38) = 7.1664, *p* = 0.0023, η^2^ = 0.27]. The Tukey HSD *post hoc* analysis revealed as significant the difference between S_BIO and S_Tri_75 (*p* = 0.0016), whereas the difference between S_BIO and S_Tri_25 does not rise the significance (*p* = 0.188).

**FIGURE 5:**
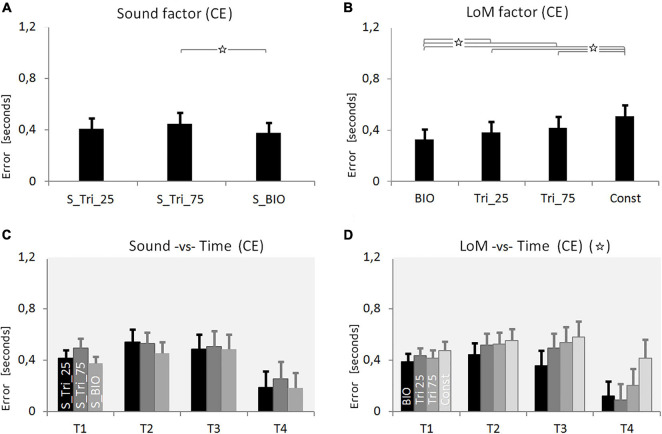
Results of Experiment 3. **(A)** Constant Error (CE) for the three Sound conditions: S_Tri_25, S_Tri_75, S_BIO (pitch envelopes are presented in Figure 2 and described in the text). **(B)** CE for the four Laws of Motion (LoM): Biological velocity profile (BIO), Triangular velocity profile with a peak of velocity at 25% of the trajectory (Tri25), Triangular profile with a peak at 75% (Tri75), Constant velocity profile (Const). **(C)** Interaction between Sound and Time main factors. Each group of bars refers to a different Time condition (T1 = 0.5 s, T2 = 1.0 s, T3 = 1.5 s, T4 = 3.0 s); in each group, bars respectively presents the Constant Error value of the three Sound conditions: S_Tri_25, S_Tri_75, S_BIO (respectively black, dark-gray and light-gray color). **(D)** Interaction between LoM and Time main factors. For each Time conditions (T1 = 0.5 s, T2 = 1.0, T3 = 1.5, T4 = 3.0) bars represent the Constant Error of the LoM conditions (from the black to the light-gray bar, respectively: BIO, Tri25, Tri75, Const). For all of the charts: *Y*-values are in seconds, error bars represent standard errors of the mean, and stars indicate significant statistical differences (when the star is in the headline, please refer to the text for details regarding the statistical differences between conditions).

The LoM factor was confirmed as statistically significant [*F*(3,57) = 27.736, *p* < 0.00001, η^2^ = 0.59]. As shown in Figure 5, the BIO kinematics condition guaranteed the most accurate results, and the Constant kinematics still produced the worst estimations. A *post hoc* Tukey HSD analysis confirmed that the BIO condition was statistically different from any other condition (*p* = 0.039, *p* = 0.0003, *p* = 0.0001 respectively *versus* Tri_25, Tri_75 and Const). The same *post hoc* analysis showed as significant the difference between the Const condition and all the other three Laws of Motion (for all cases *p* < 0.0005).

Also the Time factor appeared statistically significant [*F*(3,57) = 4.0787, *p* = 0.011, η^2^ = 0.18]. The distribution of the CE over Time, graphically presented in Figure 5, revealed the increasing of the estimation error from T1 to T3, and then a decrease in T4 where we found the smallest CE values – as already found in previous experiments. The *post hoc* Tukey HSD analysis revealed as significant the difference between T4 and all the other Time conditions.

Only the interaction between Law of Motion and Time appeared significant [*F*(9,171) = 6.8060, *p* < 0.00001, η^2^ = 0.09]. As found in both previous experiments, the differences in accuracy among the four LoM conditions decrease as the motion duration decrease (Figure 5D). A *post hoc* Tukey HSD analysis stated that only in T4 the differences among all the LoM conditions were significant (*p* < 0.00003). The BIO kinematics, instead, appeared significantly more accurate than other kinematics both in T3 and T4; no significant differences were found in T1 and T2. The ANOVA analysis on the Variable Error revealed as significant only the Time main factor [*F*(3,57) = 26.195, *p* < 0.00001, η^2^ = 0.57]. As resumed in Table 3, the VE monotonically increased from T1 to T4; the Tukey HSD *post hoc* analysis showed as significant the difference between T1 and T3 (*p* = 0.028) and between T4 and all other conditions (*p* < 0.0002).

**TABLE 3:** Results of Experiment 3 – Constant Error (CE) and Variable Error (VE) for the three main factors: Sound, Time and Law of Motion (LoM).

Sound:	S_Tri_25	S_Tri_75	S_BIO
	CE = 0.41 ± 0.36	CE = 0.44 ± 0.38	CE = 0.38 ± 0.35
	VE = 0.47 ± 0.14	VE = 0.48 ± 0.14	VE = 0.46 ± 0.12

LoM:	BIO	Tri_25	Tri_75	Const
	CE = 0.33 ± 0.35	CE = 0.38 ± 0.36	CE = 0.42 ± 0.37	CE = 0.51 ± 0.39
	VE = 0.45 ± 0.13	VE = 0.48 ± 0.13	VE = 0.47 ± 0.13	VE = 0.47 ± 0.14

Time:	T1	T2	T3	T4
	CE = 0.43 ± 0.28	CE = 0.51 ± 0.40	CE = 0.49 ± 0.52	CE = 0.21 ± 0.56
	VE = 0.29 ± 0.14	VE = 0.34 ± 0.10	VE = 0.36 ± 0.10	VE = 0.50 ± 0.12

*All values are given in seconds (mean value ± standard deviation).*

The CE and VE values obtained in the third experiment are summarized in Table 3. The table allows the comparison of the mean values obtained in each experimental condition. Consistently with the initial speculation, the comparison among the three auditory conditions shows that the S_BIO Sound constitutes the most favorable condition, although the other sounds appeared simpler.

The BIO kinematics still demonstrated supporting the best performance, as already found in previous experiments. Moreover, and consistent with previous results, participants’ responses presented the worst accuracy in estimating the Constant motion. Finally, the differences between conditions appear more pronounced in the longest experimental condition (T4 = 3 s), as already found in previous experiments.

Interestingly, despite the adoption of sound profiles analogous to the velocity profiles, Sound and Law of Motion’s main factors still appeared unrelated. Analyses of the data show no statistically significant difference between the incoherent and coherent conditions (i.e., conditions in which the visual and auditory stimuli are characterized by the same profile): CE_*BIO*__+__*S–BIO*_ = 0.29 ± 0.08; CE_*Tri*__25__+__*S–Tri*__25_ = 0.41 ± 0.09; CE_*Tri*__75__+__*S–Tri*__75_ = 0.48 ± 0.09; CE_*overall*_ = 0.41 ± 0.08 (mean values ± stand.dev., in seconds).

Summarizing the main results of the three tests: The presence of a concurrent sound clearly influences our perception of a visual motion. Compared to the silent condition, the presence of a constant sound generates a greater error in the estimates of duration. Instead, the presence of more complex sound profiles, like increasing and/or decreasing sounds, improves the perception performance. The condition that guarantees the best performance is the bell-shaped pitch profile. Statistical analyses show non-significant interaction between Sound profiles and the Laws of Motion.

## General Discussion

The present study investigates the bimodal perception of motion duration (visual and auditory). The results of the conducted tests provide evidence that the presence of an auditory stimulus affects the unified perception of motion. Moreover, the structure of each percept appears able to define, and bias, the unified perception independently.

A large body of research demonstrates that multimodal perception improves the quality of unified perception, and supports more performing and effective actions (Frassinetti et al., 2002; Bahrick et al., 2004; Teder-Sälejärvi et al., 2005; Bult et al., 2007). Unified perception arises from the non-simple composition of information coming from different perceptual channels. However, how the perceptual system combines the different pieces of information remains a matter of debate. It is a common experience that the coherence –or incoherence– among the incoming stimuli could originate, in specific conditions, an overall perception that does not correspond to the physical reality [for instance: in the ventriloquism effect (Slutsky and Recanzone, 2001; Morein-Zamir et al., 2003); in spatial or temporal illusions (Mateeff et al., 1985; Kitajima and Yamashita, 1999; Shams et al., 2002; Watkins et al., 2006), or alteration of other attributes (Kitagawa and Ichihara, 2002; Shams et al., 2002, 2005)]. The characteristics of each concurrent percept might be able to differently define the overall perception.

The present study investigates the effect of a concurrent sound on the visual perception of motion, in a duration reproduction paradigm. The two percepts that compose each stimulus have the same duration and are consistent with respect to the temporal estimation task; each percept, however, has a different structure. Four different laws of motion and nine different sound profiles were tested, to investigate whether the presence of sound alters the visual perception of motion and whether the structure of individual stimuli can bias the unified perception. The outcomes of the three tests provide interesting answers to both questions. In the subsequent paragraphs, we analyze the elements that answer the central questions of this research. Hereafter, we present some secondary results found in the collected data.

First, we find in the three experiments a systematic overestimation of the motion duration. This expected result is in agreement with previous studies, which explain this effect as consequence of the presence of motion within the stimulation. Previous works investigating the (unimodal) visual perception of motion, established a relation between the motion of the visual target and the dilation of the perceived duration (Brown, 1931, 1995; von Holst, 1954; Wolpert et al., 1995; Kanai et al., 2006; Kaneko and Murakami, 2009). To account for this same effect Brown, and subsequently Poynter and colleagues, proposed that the perceived duration may be determined by the amount of change experienced by the observer (Poynter, 1989; Brown, 1995). Rovee-Collier (1995) emphasized the active role of perception and proposed that time perception may vary according to the amount of cognitive processing performed by the observer during a given interval. Moreover, the analysis of the results also reveals a trend in all three tests, in which the CE has a minimum in correspondence to both T1 and T4 durations. The most plausible interpretation of this finding is based on the action of two different superposed effects, the presence of which emerges from participants’ responses, and causing the reduction of CE toward shorter durations, and toward longer durations - thus generating the trend present in the three tests. The first factor acts at the shorter durations, notably in T1 in which participants’ responses appear “markedly stereotyped” when compared to the longer durations (mean CE values in T1 is equal to 0.43 ÷ 0.47 s, in the three experiments). Previous literature suggests that sub-second stimuli (T1) are processed differently and by different structures than super-second stimuli (Lewis P. A. and Miall, 2003; Lewis P. and Miall, 2003; Mauk and Buonomano, 2004). The second factor, however, acts at longer durations and originates from the perception of motion. Indeed, research has shown in many experimental tests that the presence of motion induces overestimation, and also that its magnitude is proportional to the perceived velocity (Brown, 1995; Kaneko and Murakami, 2009). As consequence, the decrease of the target velocity from T1 to T4 originates a reduction of the overestimation magnitude from T1 to T4. The sum of these two effects, can easily explain the trend that emerges in T1–T2–T3–T4. Whatever, further and specific research on this subject is needed to confirm these hypotheses and to understand the mechanisms on which they rely.

Second result: concerning the perception in multimodal conditions, a large body of literature emphasizes the auditory temporal processing dominance over other perception channels (Repp and Penel, 2002; Grahn et al., 2011). Contrary to these previous studies, in the present study we did not find any dominance effect of sound on visual perception. Moreover, in Experiment 3 we verified that neither a coherent nor an incoherent sound alters the advantage offered by the biological law of motion (visually perceived).

Finally, contrary to expectations, the analyses show the absence of an effect due to consistency (or inconsistency) between the profile of visual stimulus and the profile of auditory stimulus. To account for this result, we can postulate the hypothesis that the two perceptual channels operate independently in the construction of whole perception, in accordance with the results obtained from the three tests. However, to correctly identify the origin of this effect, and to improve our knowledge of sensory integration mechanisms with consistent/inconsistent percepts, new and specific research is needed.

### Biological Motion Provides the Most Effective Support to the Perception, Constants Motion the Worst

Our results confirm the supportive role of the Biological kinematics already reported by Pozzo et al. (2006) and Carlini et al. (2012): the Biological law of motion enhances the perception-processing of the stimulus. Previous literature attributes this result to the availability of an internal model of this specific kinematics. This condition results in better accuracy and higher precision in the participants’ estimations. Interestingly, the present results also show that the Constant motion originates the worst performances, compared to any other kinematics. This result is in agreement with what Matthews obtained in his experiments with rotating figures (Matthews, 2011).

Moreover, the analysis of the interaction between Laws of Motion and Time durations reveals that the difference in accuracy between the best condition (Biological kinematics) and the worst condition (Constant kinematics) significantly increases as the motion duration increases; this effect is present in all three experiments (see Figures 3D, 4D, 5D). For all the motion profiles, from T1 to T4 the corresponding CE initially increases and then decreases. In T4, Biological motion originates a largely reduced CE, and the reduction of CE compared to shorter durations is very pronounced. Conversely, the trend of CE for the Constant motion appears to be affected minimally by stimulus duration. In the longer durations (T3, T4) the differences between the laws of motion appear well defined, whereas in the shortest duration (T1) participants’ response appears “stereotyped” for any law of motion. Difficulty in processing a too short and fast stimulus appears to be the more probable explanation for this stereotyped response.

### A Concurrent Sound Modifies the Perception of Motion

The present results show that the presence of a sound significantly influences the whole perception of motion. More interestingly, different sound profiles influence perception in different ways. The results of the three experiments coherently show that the perceived duration of the same visual motion is estimated as longer or shorter, depending on the structure of the concurrent auditory stimulation. Moreover, the analysis of the interaction between Sound profiles and Time duration reveals the presence of the same trend previously reported between the Laws of Motion and Time duration. From T1 to T4, the duration estimation error initially increases and then decreases. Additionally, in the shorter duration condition (T1 = 0.5 s), the subjects’ responses appear stereotyped, and centered on the CE value = 0.4 s, for any type of auditory stimulus.

The BIO bell-shaped profile originated the best result, for both the auditory and visual perceptual channels. Conversely, the constant stimuli originated the worst result in both channels, although they apparently constitute the easiest condition. The antithetical results obtained from these two specific conditions, and the intermediate results obtained from the other conditions, lead to explain these results by hypothesizing the existence of a similar mechanism for the two perceptual channels, in which the structure of the stimulus profile plays a primordial role. Some features of the profiles, lacking or absent in the constant profiles and present in different quantity and quality in the other conditions, could support the perception of stimuli and the duration estimation. In the shorter duration condition, in which the stimulus is more compressed in time, this same feature might be difficult to find, losing effectiveness. Future research will be required to verify this hypothesis.

**FIGURE 6:**
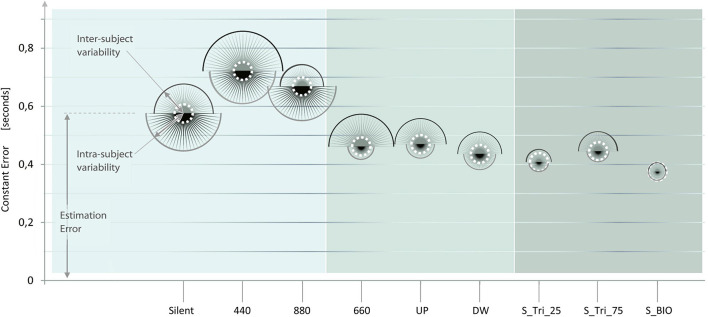
Effect of the Sound structure on the accuracy and precision time estimation. The results of the three experiments are presented in sequence, from left (Experiment 1, light gray) to right (Experiment 3, darker gray). The *x*-axis presents the nine sound conditions presented to the participants, from the easier sound (on the left) up to the most complex sound profile (on the right). For each Sound condition, the y-coordinate of each balloon-center represents the Constant Error value (i.e., the inverse of accuracy). Two half-circles compose each balloon and represent respectively: the inter-subject variability (upper half-circle), and the intra-subject variability (lower half-circle). Thus, the sizes of each balloon represent the inverse of the participants’ estimation precision. The lower and the smaller is a balloon, and the more accurate and precise are the answers for the corresponding sound condition. Balloons’ diameters are linearly proportional to the standard deviation of the subjects’ responses, and not-in-scale with the Constant Error. The white dotted line represents the distribution in the “BIO” condition, reported as a reference in the nine sound conditions. The representation of the experiments side by side is purely qualitative and no definitive comparisons can be assumed between experiments.

Figure 6 presents an overview of the nine sound conditions, in a single qualitative diagram. The Sound conditions of Experiment 1, 2, and 3, are presented on the horizontal axis, from the easier sound profile (on the left-hand) up to the most complex and variable sound profile (on the right-hand). For each sound condition, a balloon indicates the accuracy and precision mean values of the participants’ responses. The *Y*-values of the balloons’ center represents the mean CE values; consequently, a lower balloon denotes a higher accuracy for the corresponding sound condition. Each balloon is constituted by two semicircles, representing the dispersion of the estimations (i.e., the inverse of precision). The upper semicircle represents the inter-subject variability; the lower semicircle represents the intra-subject variability. Thus, smaller balloons denote higher precision and coherence for the corresponding sound condition. The dashed white circle reproduces the values of the BIO condition, to facilitate the comparison of the different conditions.

Observing the elevation and the size of the balloons, from left to right, a trend seems to emerge. This trend suggests the improvement of the participants’ estimates – both in accuracy and in precision – from the constant to the bell-shaped sound condition. A possible explanation proposes that some specific feature (e.g., the points of variation) of more complex stimuli may enhance their perception and/or processing (Libet et al., 1979; Fendrich and Corballis, 2001). The comparison among the results of the three experiments is only indicative. Future research should investigate whether the differences among the inter-experiment conditions are statistically significant, and the validity of the emerging trend.

## Conclusion and Future Perspectives

The results of the present study show that the presence of a sound modifies the perception of motion. Moreover, the specific structure of each percept – through the sensory integration – influences the whole perception of duration. Results also show that constant stimuli generated the largest errors in both auditory and visual inputs, and a possible trend according to which performance would improve with more complex stimuli. Future research should be conducted to study what perceptual cues are used in temporal estimation, both in visual and auditory channels. Future experiments could also use the point-light walker, comparing congruent and incongruent conditions of motion and sound, to further investigate perceptual integration and biological movement.

## Data Availability Statement

The datasets presented in this study can be found in online repositories. The names of the repository/repositories and accession number(s) can be found below: https://data.mendeley.com/datasets/mf4v7m7gjr/2.

## Ethics Statement

Ethical review and approval was not required for the study on human participants in accordance with the local legislation and institutional requirements. The patients/participants provided their written informed consent to participate in this study.

## Author Contributions

AC was in charge of the theoretical framework, conducted the experiments, analyzed the results, and wrote the manuscript. EB was in charge of the theoretical framework, and of the analysis of results. Both authors contributed to the article and approved the submitted version.

## Conflict of Interest

The authors declare that the research was conducted in the absence of any commercial or financial relationships that could be construed as a potential conflict of interest.

## Publisher’s Note

All claims expressed in this article are solely those of the authors and do not necessarily represent those of their affiliated organizations, or those of the publisher, the editors and the reviewers. Any product that may be evaluated in this article, or claim that may be made by its manufacturer, is not guaranteed or endorsed by the publisher.
